# Proposed Definition of Experimental Secondary Ischemia for Mouse Subarachnoid Hemorrhage

**DOI:** 10.1007/s12975-020-00796-y

**Published:** 2020-03-09

**Authors:** Jasper Hans van Lieshout, Serge Marbacher, Sajjad Muhammad, Hieronymus D. Boogaarts, Ronald H. M. A. Bartels, Maxine Dibué, Hans-Jakob Steiger, Daniel Hänggi, Marcel A. Kamp

**Affiliations:** 1 Department of Neurosurgery, Medical Faculty, Heinrich-Heine-University Düsseldorf, Moorenstraße 5, D-40225 Düsseldorf, Germany; 2 Department of Neurosurgery, Radboudumc Medical Center, Geert Grooteplein Zuid 10, 6525 GA Nijmegen, the Netherlands; 3 Department of Neurosurgery c/o Neuro Research Office, Kantonsspital Aarau, Tellstrasse 1, 5001 Aarau, Switzerland

**Keywords:** Experimental subarachnoid hemorrhage, Delayed cerebral ischemia, Outcome parameter

## Abstract

Inconsistency in outcome parameters for delayed cerebral ischemia (DCI) makes it difficult to compare results between mouse studies, in the same way inconsistency in outcome parameters in human studies has for long obstructed adequate comparison. The absence of an established definition may in part be responsible for the failed translational results. The present article proposes a standardized definition for DCI in experimental mouse models, which can be used as outcome measure in future animal studies. We used a consensus-building approach to propose a definition for “experimental secondary ischemia” (ESI) in experimental mouse subarachnoid hemorrhage that can be used as an outcome measure in preclinical studies. We propose that the outcome measure should be as follows: occurrence of focal neurological impairment or a general neurological impairment compared with a control group and that neurological impairment should occur secondarily following subarachnoid hemorrhage (SAH) induction compared with an initial assessment following SAH induction. ESI should not be used if the condition can be explained by general anesthesia or if other means of assessments sufficiently explain function impairment. If neurological impairment cannot reliably be evaluated, due to scientific setup. Verification of a significant secondary impairment of the cerebral perfusion compared with a control group is mandatory. This requires longitudinal examination in the same animal. The primary aim is that ESI should be distinguished from intervention-related ischemia or neurological deficits, in order establish a uniform definition for experimental SAH in mice that is in alignment with outcome measures in human studies.

## Background

Early brain injury (EBI) and delayed cerebral ischemia (DCI) are the two main determinants for survivors’ outcome after aneurysmal subarachnoid hemorrhage (aSAH). DCI and EBI are well defined for clinical use and have been studied extensively in order to treat EBI-related symptoms, determine the prognosis of aSAH patients, reduce the incidence of DCI, and improve functional outcome after aSAH. Mice were the latest species that was introduced into experimental SAH research in 1999 [20]. But the number of studies in mice has steadily increased during the last two decades and has—together with rat and rabbit models—become one of the most commonly used species in preclinical SAH research [21]. Various pharmaceutics showed promising results in in vivo animal studies. However, despite the high number of animal studies, different therapeutic approaches, and numerous analyzed pharmaceutics, standard therapies of DCI have barely changed and oral nimodipine remains the only drug to improve neurological outcome in aSAH patients.

In response to unsatisfactory translational results, guidelines for a standardized reporting of animal experiment have been implemented and meta-analysis of existing data has been proposed in order to detect for sources of bias and better estimate effect sizes [13, 14, 19, 32]. Despite a reduction of the species used in SAH animal models, diversity of the experimental setups has not decreased over time. The variety of experimental setups including the model (single-, double-injection model, amount of blood injected etc.) and the lack of standardization of models was considered as one reason for the disappointing translation of basic SAH research [18]. Translational difficulties might also stem from interspecies differences such as the clearance rate of subarachnoid blood or the amount of collaterals in the animal brain [17, 23]. As a result, pathophysiological processes important in humans might not have a similar relevance in an experimental setting. Moreover, DCI may have also been missed due to a lack of longitudinal observation or clear definition for DCI in experimental models [12]. This inconsistency in outcome parameters makes it difficult to compare results between studies, in the same way inconsistency in outcome parameters in human studies has for long obstructed adequate comparison of results [37]. The absence of established definition for DCI may thus in part be responsible for failed translational results [12]. The present article aims to propose standardized definitions for DCI in experimental mouse models for aSAH that can be used as outcome measures in future animal studies.

## Methods

We used a consensus-building approach analog to the method used by Vergouwen et al. [37]. At the start of the study, two authors (M.K. and J.V.L.) developed the idea to propose standardized definitions for DCI in experimental models for SAH. Both authors subsequently proposed a draft manuscript containing definitions of DCI for mouse models in experimental SAH research. We identified and contacted a multidisciplinary group of experts in the field of experimental SAH research and invited them to help shape the standardized definitions for DCI in mouse models for experimental SAH. The draft manuscript was sent to all coauthors. We implemented the suggestions of the coauthors and send the manuscript back to all coauthors. M.K. and J.V.L. mediated this process and repeated it three times until a consensus was reached between all authors.

## Considerations for the Definition

### Delayed Cerebral Ischemia

The definition of DCI in a clinical setting has been described by Vergouwen et al. as:

“The occurrence of focal neurological impairment (such as hemiparesis, aphasia, apraxia, hemianopia, or neglect), or a decrease of at least 2 points on the Glasgow Coma Scale (either on the total score or on one of its individual components [eye, motor on either side, verbal]). This should last for at least 1 hour, is not apparent immediately after aneurysm occlusion, and cannot be attributed to other causes by means of clinical assessment, CT or MRI scanning of the brain, and appropriate laboratory studies.” [37].

This definition is not applicable in experimental SAH due to multiple reasons.

1. The outcome parameters in experimental mouse models should mimic their human counterparts [31, 37]. But there are two main reasons why we cannot apply this definition directly to experimental SAH in mice. Excluding other possible causes of neurological impairment with CT or MRI scanning of the brain and appropriate laboratory testing is not feasible due to logistical and practical reasons.
2. DCI in humans usually does not occur before the third day after ictus. In experimental SAH, the phenomenon of DCI can present itself much earlier, for mice as early as 6 h after induction of SAH [10, 11]. Due to animal welfare and regulatory reasons, researchers always induce experimental SAH under general anesthesia. In our experience, adequate neurological assessment and differentiation between SAH- and anesthesia-induced neurological impairment is not possible until 12 to 24 h after SAH induction [10]. Because of the sedation, a definition based purely on clinical features might result in either over- or underestimation of the true incidence of DCI, since the mice awake from anesthesia within the DCI period, and differentiation between clinical deterioration due to DCI and EBI is challenging.
3. Many studies try to model DCI following experimental SAH. However, as stated above, it remains questionable if and to what extend animal and—in particular—mouse models resemble DCI and if DCI exists following experimental SAH. Therefore, in our view, the term “delayed cerebral ischemia/DCI” should not be used in connection with an experimental setup. Instead, we propose to use the term “experimental secondary ischemia or ESI” for those pathophysiological and clinical reactions which should resemble DCI in experimental SAH and which occur secondary following early brain injury.

### What Kinds of Measures Are Essential for a Definition of ESI in Mice?

1. Neurological testing for small ischemia in mice is difficult due to the poor sensitivity of neurocognitive testing. Mice have a total brain volume of about 500 mm^3^ and may have 10–30-mm^3^ infarctions by a total brain volume without any detectable motor impairment [16]. This may contribute to the difficulties faced in SAH research and standard of reporting. Researchers may also miss smaller cortical cerebral ischemia as they are mostly not clinically manifested in mice. A good example for this is the distal pMCAO mouse model [24, 25, 30]. Also, deficits caused by SAH induction itself may be severe enough to mask secondary neurological deficits. However, as clinical deterioration is an integral part of the DCI definition, secondary focal or global neurological impairment should be part of the ESI definition.
2. The term “delayed cerebral ischemia” implies that ischemia is responsible for focal and neurological impairment in aSAH patients. Since not all SAH models may effectively detect neurological impairment, detection of any kind of significant changes in cerebral perfusion or infarction should be an integral part of a definition of ESI. If neurological examination cannot reliably be performed due to the scientific setup, it is critical to ascertain whether any ischemia develops in a delayed manner, which may qualify for DCI.
3. Although not explicitly stated in the clinical definition by Vergouwen et al., the term DCI refers to a secondary clinical deterioration [37]. DCI should be separated from ischemia caused by perfusion impairment closely following aneurysm rupture and from intervention associated cerebral ischemia: Aneurysm rupture typically induces a steep increase of the intracranial pressure (ICP) and a massive decrease of the cerebral perfusion and may subsequently lead to disruption of neuronal signaling and to a generalized or focal hypoxic injury [35]. In experimental SAH, perfusion impairment associated with SAH induction and related to interventions should clearly be separated from DCI. Again, primary (e.g., EBI or intervention-related ischemia) and secondary neurological impairment cannot reliably be distinguished in mouse models due to general anesthesia and earlier onset of DCI. However, a secondary—and not a primary—impairment of neurological function and cerebral perfusion is an important feature of DCI and necessary to differentiate EBI and DCI. We therefore recommend that neurological impairment or impairment of the cerebral perfusion should occur secondarily after SAH induction and requires longitudinal assessment of the same animal [10, 11]. Initial assessment should document deficits 12 to 24 h after SAH induction and serial exams should follow in order to detect any exacerbation [10].
4. As in humans, ESI should ideally be a diagnosis per exclusionem. However, since other factors such as edema, hydrocephalus, metabolic causes, or iatrogenic lesions caused by SAH induction are difficult to distinguish, it is inherently difficult to know for sure whether clinical deteriorations can be truly attributed to ESI. This introduces a possible source of overestimation. Only in the case that other assessments (e.g., imaging, laboratory, or autopsy results) or induction of SAH itself sufficiently explains focal or global function impairment must ESI not be taken in to account.
5. Clinical and pathophysiological responses following aSAH should be compared with at least one adequate control group (ideally a sham and saline control group). By using suitable controls, such as a control saline injections, effects of subarachnoid blood can be differentiated from other effects caused, e.g., by injection or temporary filament occlusion. As control groups are—of course—not part of the clinical DCI definition, we consider adequate control group not as a part of definition of ESI but of good scientific practice.

### Neurological Impairment

Clinical examination for stroke and aSAH patients is more or less standardized [5, 8, 29, 33]. A standardized neurological evaluation is the prerequisite for clinical grading and prediction of clinical outcome [5, 8, 33].Various versions of scores for neurological assessment in mice are used, including a Modified Mouse Coma Scale, however no common scoring system for injury severity that has been widely adopted for animals based on a brief neurological examination, like the Glasgow Coma Scale in human patients with aSAH [1–4, 6, 7, 10, 26, 28, 36]. Neurological assessment of mice in experimental SAH derives from other species and stroke research [4, 7, 28]. Next to behavioral tests, some well-established motor function tests, such as the tape removal test or the rotarod test, are used in experimental mouse SAH [9, 15, 22, 26, 34]. However, scores for neurological evaluation assessing motor and sensory function are more common: These scores derive from rat models in translational stroke research and are adapted for their use in experimental mouse models. Recommendations for a basic neurological assessment of mice in experimental SAH might increase comparability between studies, but are beyond the scope of the present manuscript. We recommend using an established score for a basic neurological assessment, which allows for standardized neurological assessment and comparability between different experimental groups.

### Assessment of Cerebral Perfusion Impairment and Cerebral Infarction

As previously stated, detection of any kind of cerebral infarction and/or cerebral perfusion deficit should be an integral part of a definition of experimental secondary ischemia. However, there is a widespread of substitutes and definitions for vasospasms and perfusion impairment throughout the literature on experimental mouse SAH. Standardization of assessing cerebral vasospasms and perfusion impairment is beyond the scope of this manuscript. However, there is a disconnection between large artery vasospasms and clinical outcome in humans. In our view, evaluation of cerebral perfusion impairment or detection of infarctions should therefore be preferred to detection of cerebral vasospasms. Ideally, cerebral perfusion would be measured without sedation or anesthesia but due to animal welfare and regulatory reasons, the initial perfusion measurement during SAH induction will always need to be performed under general anesthesia. Moreover, as disconnection between large artery vasospasms and clinical outcome in humans might be one reason for the translational problem, assessment of cerebral perfusion should ideally not only focus on verification of large artery vasospasms but also quantify the cerebral perfusion itself. Neuroimaging and brain perfusion assessments should ideally be used to discern injury early after SAH induction and serve as comparison for follow-up to identify secondary ischemia. Clearly, neuroimaging in experimental SAH is not routinely used and its impracticality makes it rather not advisable.

## Proposed Standardized Definitions for “Experimental secondary Ischemia” in Experimental Mouse Models for Aneurysmal Subarachnoid Hemorrhage

### Proposed Definition of ESI in Experimental Mouse SAH

1. Occurrence of focal neurological impairment (as assessed by standard score to assess neurologic performance in mice after SAH), or a general neurological impairment (lethargic or comatose condition) compared with a control group

and

(2) Neurological impairment should occur secondarily following SAH induction compared with the first possible assessment following SAH induction

and

(3) ESI should not be taken in to account if the condition can be explained by general anesthesia or if other means of assessments (confirmed by autopsy results) sufficiently explain focal or global function impairment

or

(4) If 2 does not apply, due to scientific setup. Verification of a significant secondary impairment of the cerebral perfusion (e.g., cerebral hypoperfusion of infarction demonstrated by follow-up imaging or histopathology) compared with a control group is mandatory. This requires longitudinal examination in the same animal.

## Implications and Generalization of ESI to Other Species

A recent literature review suggests that animal models, with the exception of primates, do not fully recapitulate the progression of DCI in humans [27]. Especially delayed/secondary neurological deficits were not detected in most mice, dog, rat, or rabbit models, but the incidence in primate models was similar to the incidence of delayed cerebral ischemia in humans. The majority of animal models could not faithfully reproduce a pathophysiological situation, which was severe enough to cause infarction, regardless of the method and species [27]. Similarly, the mean overall mortality rate in experimental mouse models of aSAH is significantly lower than the reported human case fatality [11].

It could be argued that therefore is a need for the development of new animal models that more faithfully resemble the human condition for both case fatality and DCI. The elastase-hypertension model is one such an attempt to more faithfully reproduce the human condition. However, this model is not without its own problems. The unpredictability of aneurysm rupture and elastase-induced changes to the arterial wall may complicate the study of DCI in this model. Also, other than that the mechanism of rupture seems to be more similar, there are no definitive indications that such a model will be able to better recapitulate the progression of DCI in humans.

In parallel, it might be useful to better understand what exactly happens in our models. It is therefore important to establish a uniform terminology in order to address the inconsistency in outcome parameters, which has complicated adequate comparison of results between studies. Certain models may turn out to more effectively reproduce ESI than others. Although primarily aimed at the mouse model, the same definition of ESI might be applied to other animal models of experimental SAH.

The present definition dispenses a timely definition of DCI and EBI. As previously mentioned, the timely classification between EBI and DCI is difficult in experimental SAH, has not been really helpful in the clinical setting, and has very limited value in the experimental setting. Moreover, it remains questionable if EBI and DCI are pathophysiologically linked and if there is a unifying link between EBI and DCI in either human or experimental SAH. A division in primary and secondary ischemia might be better transferable. This terminology links primary ischemia to the perfusion deficits and hypoxia directly caused by the pathophysiological changes immediately following aneurysm ruptures and secondary ischemia to all brain perfusion impairments following (partial) recovery of primary ischemia. From our impression, primary ischemia seems to be highly comparable between different species and involves similar pathophysiological mechanisms including severe cerebral perfusion impairment in parallel to a massive increase of the ICP and a disruption of neuronal signaling. In contrast, occurrence, time-point, and mechanisms of secondary ischemia seem to be much more heterogeneous and likely significantly vary between different species.

It remains to be tested if therapeutic interventions that effectively reduce the occurrence of ESI are more predictive of translational results in humans. We recognize that the establishment of a definition for ESI requires a broad international consensus and that trying to introduce a clinically relevant outcome measure is challenging. However, we hope that ESI aids the clinical relevance and predictive value of experimental mouse models of SAH for DCI. Future efforts should systematically investigate to what extent aSAH animal models resemble the human condition and how parameters such as the experimental model, animal species, anesthesia, or the genetic background affect EBI- and DCI-related pathophysiological responses.

## Conclusion

We propose a standardized definition for experimental secondary ischemia (ESI) in experimental mouse SAH analog to the DCI in humans which can be used for future experimental SAH research in mouse models. The primary aim is that ESI should be distinguished from intervention-related ischemia or neurological deficits.

We hope to spark a broader discussion about the comparability of definitions used in clinical practice and in a laboratory setup. A common language (standardized definitions) of additional core characteristics and endpoints of preclinical SAH research, based on common data elements for data collection and reporting, will be important to improve quality of SAH research and translational success.
